# Evaluating the cost of simplicity in score building: An example from alcohol research

**DOI:** 10.1371/journal.pone.0294671

**Published:** 2023-11-27

**Authors:** Valentin Rousson, Bastien Trächsel, Katia Iglesias, Stéphanie Baggio

**Affiliations:** 1 Division of Biostatistics, Center for Primary Care and Public Health (Unisanté), University of Lausanne, Lausanne, Switzerland; 2 School of Health Sciences Fribourg (HEdS-FR), HES-SO University of Applied Sciences and Arts of Western Switzerland, Fribourg, Switzerland; 3 Institute of Primary Health Care (BIHAM), University of Bern, Bern, Switzerland; 4 Laboratory of Population Health (#PopHealthLab), University of Fribourg, Fribourg, Switzerland; Sunway University, MALAYSIA

## Abstract

Building a score from a questionnaire to predict a binary gold standard is a common research question in psychology and health sciences. When building this score, researchers may have to choose between statistical performance and simplicity. A practical question is to what extent it is worth sacrificing the former to improve the latter. We investigated this research question using real data, in which the aim was to predict an alcohol use disorder (AUD) diagnosis from 20 self-reported binary questions in young Swiss men (n = 233, mean age = 26). We compared the statistical performance using the area under the ROC curve (AUC) of (a) a “refined score” obtained by logistic regression and several simplified versions of it (“simple scores”): with (b) 3, (c) 2, and (d) 1 digit(s), and (e) a “sum score” that did not allow negative coefficients. We used four estimation methods: (a) maximum likelihood, (b) backward selection, (c) LASSO, and (d) ridge penalty. We also used bootstrap procedures to correct for optimism. Simple scores, especially sum scores, performed almost identically or even slightly better than the refined score (respective ranges of corrected AUCs for refined and sum scores: 0.828–0.848, 0.835–0.850), with the best performance been achieved by LASSO. Our example data demonstrated that simplifying a score to predict a binary outcome does not necessarily imply a major loss in statistical performance, while it may improve its implementation, interpretation, and acceptability. Our study thus provides further empirical evidence of the potential benefits of using sum scores in psychology and health sciences.

## Introduction

Composite scores are widely used in psychology and health sciences. Guidelines are available for the development and validation of these scores, but recommendations for analytical strategies are less common [1]. Composite scores can be calculated at different levels of complexity [2]. The simplest composite score would be a sum score, in which the possible values are restricted to be either +1 or 0. In this sum score, all questions with a non-zero coefficient have the same positive weight. More sophisticated approaches to composite scores include the use of restricted value ranges (e.g., +1, -1 or 0) or linear combinations of the items. Such composite scores (“refined scores”) can be developed using logistic regression models with a gold standard as the response variable and the items as predictors. These approaches allow for unequal weighting of questions.

### Controversy on simple scores

The use of simple or refined scores has been much discussed and is still currently debated. First, it has been discussed in the context of scores obtained from factor or principal component analyses [3–7] with conflicting conclusions. While two recent studies warned that sum scores may be too imprecise for use in rigorous research applications [8, 9], another study presented an example where little was gained from the use of factor score estimates (i.e., refined scores) compared to simpler sum scores [10]. A third opinion paper also concluded that sum scores are suitable to build scores [2]. In the context of linear regression modeling, previous studies suggested that equal regression weights might be a reasonable choice [11–13], especially if predictors are standardized, with a modest loss of accuracy compared to unequal weight [11]. To our knowledge, the use of simple or refined scores was not discussed in the context of logistic regression. Further empirical investigations are therefore needed to better understand the benefits and limitations of simple or refined scores in this analytical context, as stated in recent studies [10, 13].

### Understudied perspectives

An interesting perspective that has been neglected in previous research is to identify the *cost of simplicity*. To facilitate implementation, interpretation, and acceptability, simple scores sacrifice some of the statistical performance for the sake of simplicity. If the loss of statistical performance does not appear to be substantial, this would argue in favor of using a simple rather than a refined score.

In addition, when evaluating the statistical performance of a score, it is important to consider problems of overfitting, also known as optimism, and to attempt to correct for them [14]. Overfitting occurs, for example, when a regression model includes too many predictors, but also when it is selected from a large family of candidate models, e.g., via automated variable selection [15]. Overfitting may lead to replicability issues, a critical issue in psychology and health sciences [16]. Simple scores may be less prone to overfitting than refined scores. This may be an unexplored advantage of simple scores over refined scores.

### Objective of the study

The aim of the present study was therefore to investigate and evaluate the cost of simplicity using real-life data, where the aim was to predict a diagnosis of alcohol use disorder (AUD) diagnosis from 20 self-reported binary questions. Unlike factor analysis, where a score is developed to measure a theoretical construct that is not observable, we had the advantage of having a gold standard against which to compare our predictions. It was therefore possible to objectively compare the statistical performance (including optimism) of refined and simple scores obtained by different methods.

## Materials and methods

### Design

We re-used data from a prospective cross-sectional study designed to identify an accurate screening tool for AUD [17, 18]. The study was approved by the Ethics Committee of the Canton of Vaud (no. 2017–00776). Participants signed a written informed consent for the study and an additional consent form to accept the reuse of their data in further projects. The authors did not have access to any information that could identify individual participants during or after data collection.

### Participants

Data were collected from October 2017 to June 2018 in a sample of young Swiss men. They were recruited from the Cohort Study on Substance Use and Risk Factors (C-SURF) [19]. Inclusion criteria were 1) being a French-speaking participant, 2) completing the second follow-up questionnaire (from 2016 to 2018), and 3) having a valid email (n = 2,668). Eligible participants were invited to complete the ten-question version of the Alcohol Use Disorder Identification Test (AUDIT) [20] online (1,371 respondents, response rate = 51.4%). Participants were then selected using a stratified sampling strategy: those with a high AUDIT score (≥13) and those with a low score (<13) [21]. The final sample size was 233 (total response rate = 70.6%, 68.9% in the low-strata group and 72.0% in the high-strata group).

### Diagnosis of AUD

A binary variable measured the presence or absence of AUD, assessed with a clinician-administered diagnostic interview (Diagnostic Interview for Genetic Studies (DIGS) [22]) and representing the gold standard. The DIGS has a high inter-rater agreement and a good concordance with clinical diagnoses from medical records [22]. At the time of the study, the DIGS had not been adapted to the DSM-5 criteria. To address this limitation, we replaced the DSM-IV question on legal problems (removed in DSM-5) with a question on craving (added in DSM-5). AUD was defined as at least mild (cut-off score = 2) in the previous twelve months.

### Self-reported AUD and alcohol-related consequences

A set of 20 binary questions (1 = yes/0 = no, hereafter Q1-Q20) designed to screen for AUD was used to predict the gold standard. Participants self-reported the presence or absence of the eleven DSM-5 AUD criteria [20, 23] and of nine alcohol-related consequences [20, 24, 25] in the previous twelve months. The questions are listed in S1 Table.

### Analytical strategy

The sample size was calculated for the original study purpose [17, 18]. As AUD was overrepresented in our study sample, we focused on discrimination rather than on calibration [14] when assessing the statistical performance of our scores. Score performance was measured using the area under the ROC curve (AUC) [26].

### Refined score

Our aim was to build a score that best predicted the gold standard (AUD) from the responses given to questions Q1-Q20. We fitted a logistic regression model with the gold standard as the outcome and questions Q1-Q20 as binary predictors. Coefficients were used for the score.

### Simple scores

We defined four simple scores. First, we simplified non-zero coefficients with m = 3, 2 or 1 possible digit(s) (see S1 File for details). A fourth simple score allowed zero or positive coefficients, but not negative coefficients, is called a “sum score”. This is because in our example data, all 20 predictors were designed to be positively associated with the gold standard. In such a context, having negative coefficients may undermine the acceptability of a simple score, so it is tempting to remove negative coefficients (set them to zero). This is consistent with the recommendation of Steyerberg et al. [27], who advocate “using qualitative information on the sign of the effect of predictors”.

### Methods

For both refined and simple scores, we first used maximum likelihood estimation (MLE). Then, to reduce the number of predictors in the model, we used the well-known backward elimination procedure (hereafter BACKWARD), which consists of starting with a model including all the predictors and eliminating the least significant predictor at each step of an iterative procedure. We used the Akaike criterion to select the best model [28]. We also used other more modern methods for fitting a model with many predictors with penalized maximum likelihood, i.e., the LASSO or RIDGE penalty (also called the L1 or L2 penalty, respectively), where the coefficients defining the score are shrunk towards zero [29]. The LASSO penalty sets some coefficients exactly to zero, making the resulting score more parsimonious.

Finally, the results may be too optimistic. One reason is that our scores were derived and evaluated from the same data. For BACKWARD, another reason is that we used a strict model selection. To correct for optimism, we applied a bootstrapping procedure, as described and recommended by Steyerberg et al. [30]. Note that for BACKWARD and LASSO, the resulting model did not necessarily include the same number of predictors in each bootstrap resample.

In each resample and for each method, we calculated two AUCs: one using the data from the bootstrap resample and one using the data from the original sample. Optimism was estimated as the difference between these two AUCs, averaged over the 500 bootstrap resamples.

Analyses were performed using R software. For the LASSO and RIDGE procedures, we applied the default parameters implemented in the *glmnet* library (version 4.1–3). The statistical code and dataset are available as supplementary material.

## Results

The proportion of patients with AUD was 33.5% (n = 78) (mean age = 27.00). The proportion of patients answering “yes” to the different questions ranged from 4% (Q18) to 67% (Q6). All questions were significantly positively associated with the gold standard, with odds-ratios ranging from 1.89 (for Q20) to 13.08 (for Q11), except for one question (Q20). Table 1 summarizes this information and also shows the sensitivity and specificity achieved by each question, as well as the AUC, which ranged from 0.525 (for Q20) to 0.679 (for Q4).

**Table 1:** Summary of the associations between the 20 questions and the gold standard.

Question	Proportion yes	Sensitivity	Specificity	AUC	OR	P-value
Q1	75/233 = 32%	45/78 = 58%	125/155 = 81%	0.692	5.68	< .001
Q2	150/233 = 64%	64/78 = 82%	69/155 = 45%	0.633	3.67	< .001
Q3	27/233 = 12%	20/78 = 26%	148/155 = 95%	0.606	7.29	< .001
Q4	49/233 = 21%	35/78 = 45%	141/155 = 91%	0.679	8.20	< .001
Q5	27/233 = 12%	21/78 = 27%	149/155 = 96%	0.615	9.15	< .001
Q6	155/233 = 67%	64/78 = 82%	64/155 = 41%	0.617	3.22	.001
Q7	24/233 = 10%	16/78 = 21%	147/155 = 95%	0.577	4.74	.001
Q8	77/233 = 33%	46/78 = 59%	124/155 = 80%	0.695	5.75	< .001
Q9	12/233 = 5%	9/78 = 12%	152/155 = 98%	0.548	6.61	.006
Q10	30/233 = 13%	23/78 = 29%	148/155 = 95%	0.625	8.84	< .001
Q11	19/233 = 8%	16/78 = 21%	152/155 = 98%	0.593	13.08	< .001
Q12	55/233 = 24%	31/78 = 40%	131/155 = 85%	0.621	3.60	< .001
Q13	110/233 = 47%	54/78 = 69/	99/155 = 64%	0.666	3.98	< .001
Q14	108/233 = 46%	54/78 = 69%	101/155 = 65%	0.672	4.21	< .001
Q15	56/233 = 24%	33/78 = 42%	132/155 = 85%	0.637	4.21	< .001
Q16	28/233 = 20%	15/78 = 19%	142/155 = 92%	0.554	2.60	.019
Q17	46/233 = 12%	28/78 = 36%	137/155 = 88%	0.621	4.26	< .001
Q18	10/233 = 4%	7/78 = 9%	152/155 = 98%	0.535	5.00	.022
Q19	37/233 = 16%	22/78 = 28%	140/155 = 90%	0.593	3.67	< .001
Q20	19/233 = 8%	9/78 = 12%	145/155 = 94%	0.525	1.89	.186

AUC: Area under the curve; OR: odds-ratio.

**Fig 1:**
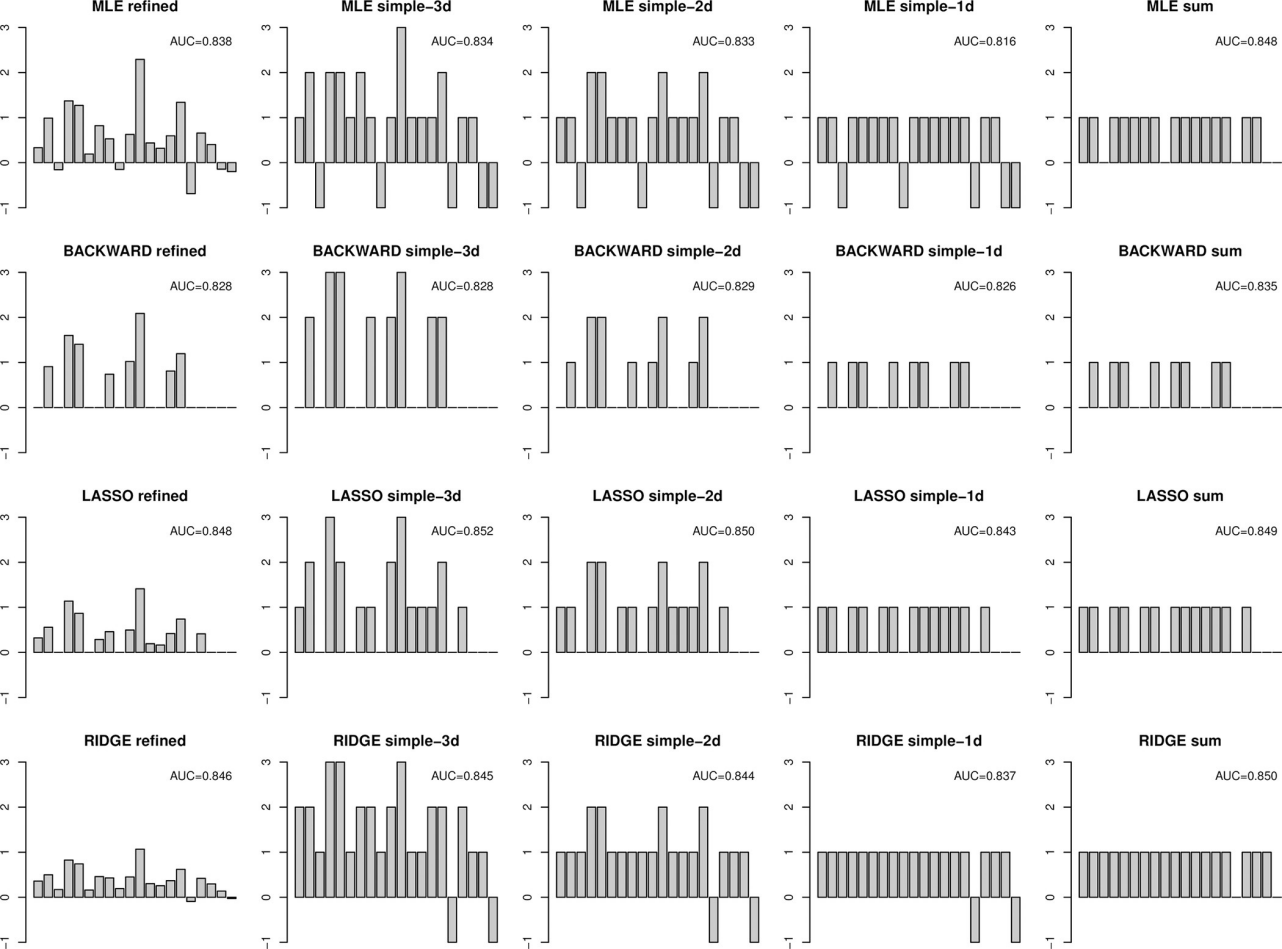
Graphical representation of the coefficients of refined and simple scores to predict the gold standard obtained using various methods, together with corrected AUCs. In each panel, the 20 bars represent the coefficients assigned to questions Q1-Q20 (from left to right). AUC: Area under the curve; MLE: maximum likelihood estimation; BACKWARD: backward elimination procedure, selection using Akaike criterion; LASSO: L1-penalized maximum likelihood; RIDGE: L2-penalized maximum likelihood. Refined score: coefficients from logistic regression; simple-3d, 2d, and 1d scores: simple scores with 3, 2, and 1 digit(s); sum score: negative coefficients set to zero and 1 digit.

The main results for the refined and simples scores and different methods are shown in Table 2. For the refined score, the AUC for MLE was 0.890, higher than the AUCs when each individual question was considered as a predictor. Using the BACKWARD procedure, the final model included eight questions (Q2, Q4, Q5, Q8, Q9, Q10, Q14 and Q15) and the AUC was 0.876. We obtained AUCs of 0.887 for LASSO (Q1, Q2, Q4, Q5, Q7, Q8, Q10, Q11, Q12, Q13, Q14, Q15 and Q17, other coefficients set to zero) and 0.881 for RIDGE. The coefficients assigned to the different questions for the four methods considered (MLE, BACKWARD, LASSO, RIDGE) are plotted in the four panels of the first column of Fig 1. None of these scores are simple since all non-zero coefficients are different from each other. It is worth noting that some coefficients were negative for MLE and RIDGE.

**Table 2:** Observed / corrected AUCs for refined and simple scores to predict the gold standard using various methods.

		Scores				
		refined	simple-3d	simple-2d	simple-1d	sum
Methods	MLE	0.890 / 0.838	0.881 / 0.834	0.878 / 0.833	0.856 / 0.816	0.864 / 0.848
		(-0.052)	(-0.047)	(-0.045)	(-0.039)	(-0.016)
	BACKWARD	0.876 / 0.828	0.873 / 0.828	0.873 / 0.829	0.866 / 0.826	0.866 / 0.835
		(-0.047)	(-0.045)	(-0.044)	(-0.040)	(-0.031)
	LASSO	0.887 / 0.848	0.886 / 0.852	0.881 / 0.850	0.870 / 0.843	0.870 / 0.849
		(-0.038)	(-0.034)	(-0.031)	(-0.027)	(-0.021)
	RIDGE	0.881 / 0.846	0.877 / 0.845	0.874 / 0.844	0.862 / 0.837	0.862 / 0.850
		(-0.035)	(-0.032)	(-0.030)	(-0.025)	(-0.012)

The optimism, which is the difference between the observed and the corrected AUC (obtained using a bootstrap procedure), is given in parentheses.

AUC: Area under the curve; MLE: maximum likelihood estimation; BACKWARD: backward elimination procedure, selection using Akaike criterion; LASSO: L1-penalized maximum likelihood; RIDGE: L2-penalized maximum likelihood.

Refined score: coefficients from logistic regression; simple-3d, 2d, and 1d scores: simple scores with 3, 2, and 1 digit(s); sum score: negative coefficients set to zero and 1 digit.

In bootstrap analyses, we estimated an optimism of 0.052, 0.047, 0.045 and 0.039 for MLE, BACKWARD, LASSSO and RIDGE, respectively. Finally, corrected AUCs were obtained by subtracting the estimated optimism from the observed AUCs, yielding 0.838, 0.828, 0.848 and 0.846, respectively (see Table 2).

The simple scores obtained with m = 3, 2 or 1 possible digit(s) are plotted in the second, third, and fourth columns of Fig 1 for the four methods. Unlike the refined score plotted in the first column of Fig 1, the non-zero coefficients of these simple scores are not all different from each other.

Observed and corrected AUCs for all simple scores are shown in Table 2. Optimism was systematically lower for simple scores than for the refined score. Among the simple scores, the optimism was also systematically lower with m = 1 than with m = 2 digits and with m = 2 than with m = 3 digits. After correcting for optimism, the best performance in terms of AUC was obtained with the simplified LASSO with m = 3 digits, with a corrected AUC of 0.852, which was even better than the refined score obtained with LASSO (with a corrected AUC of 0.848).

Finally, the fifth column of Fig 1 shows the sum scores obtained by the four methods by setting the negative coefficients to zero. For BACKWARD and LASSO, they were identical to the one-digit simple scores. These scores are sums of 15, 8, 13 and 18 questions for MLE, BACKWARD, LASSO and RIDGE, respectively. The observed and corrected AUCs for these sum scores are shown in Table 2. Optimism was even lower for sum scores than for simple scores. The corrected AUCs for sum scores were of 0.848, 0.835, 0.849 and 0.850, respectively. Except for BACKWARD, which was slightly above, the sum scores obtained via MLE, LASSO or RIDGE performed almost as well (or even better than) the refined score via LASSO (with a corrected AUC of 0.848). In particular, the sum score via LASSO with a corrected AUC of 0.849 and only 13 questions could be a good final choice for this example, as the resulting score is not only simple but also parsimonious.

It should be noted that the sum score used by Baggio et al. [17] for these data included 12 instead of 13 questions. It was not obtained as a simplified version of a refined score, but as the sum score minimizing the Akaike criterion among all 2^20–1 = 1’048’575 possible sums, achieving an observed AUC of 0.872 and a corrected AUC of 0.841.

## Discussion

In this study, we attempted to simplify and evaluate the statistical performance in terms of AUC of refined and simple scores obtained by different methods using data from alcohol research where the aim was to predict an AUD from 20 binary questions.

Among the refined score methods, the best performance was achieved by LASSO with a corrected AUC of 0. 848. The MLE method had the highest observed AUC (0.890), but it was the most optimistic method (i.e., the most prone to overfitting). However, as we only had 78 cases of AUD in our dataset, a model with 20 predictors did not follow the rule of thumb of 10 required events per predictor. This could lead to overfitting, although this rule of thumb should not be taken too strictly and has recently been questioned [31].

Among the ways of defining simple scores and related methods, a simple score with 3 digits using LASSO was the best, even better than the refined score (corrected AUC = 0.852). Other simple scores, especially sum scores, performed almost identically or even slightly better than the refined score obtained with some methods, illustrating the fact that simplifying a score does not necessarily imply a major loss in statistical performance. Indeed, the sum score had the highest corrected AUCs for MLE, BACKWARD, and RIDGE.

Overall, the more constrained the coefficients were, the less prone a method was to overfitting. Simple scores, and even more, sum scores, are less prone to overfitting than the refined score because they are less data-dependent due to the restrictions imposed on their possible values. Therefore, the simplification of a refined score does not necessarily come at the cost of sacrificing statistical performance. Such considerations were anticipated by in a previous study in the context of linear regression [11] and factor analysis [10]. The latter found that factor scores could lead to greater indeterminacy than sum scores [10]. Estimates of the former may vary from sample to sample, whereas sum scores have identical weights in all samples. Our study illustrated and confirmed this finding in the context of logistic regression.

It should be noted that the corrected AUCs provided in our data are only sample estimates of the true AUC that would be obtained by applying a score to the entire population of interest. As the confidence intervals calculated around them would largely overlap, we would not be able to conclude that the corrected AUCs for a simple score would be “significantly higher” than the corrected AUCs for the refined score. However, the reverse would also be true (the corrected AUCs for the refined score would not be significantly higher than the corrected AUCs for some simple scores) and this may already be sufficient justification for using a simple score in practice. In any case, researchers should not be discouraged a priori from striving for simplicity, as the sacrifice of statistical performance may be very small. This is in line with recent conclusions [10], which also noted that sum scores are easier to implement than factor scores.

These recommendations are based on a single, albeit real, data example, which constitutes a study limitation. In addition, the sample size was relatively small, which did not allow splitting the dataset into train and test sets. Therefore, we do not claim or advocated that it is possible to replace refined scores with simple scores in all practical cases. Rather, we encourage researchers and methodologists developing screening tools to evaluate the cost of simplicity along the lines presented here, including a correction for optimism, with their own data. Their results would provide a sound basis and some justification for deciding whether to retain a refined score or replace it with a simple score. Although researchers and statisticians have different views on the use of simple scores, there is a consensus on the need to take psychometrics seriously and to provide justification for the preferred scoring methods [2, 8, 10, 16].

## Conclusion

To conclude, our example data demonstrated that simplifying a score to predict a binary outcome does not necessarily imply a major loss in statistical performance, while potentially improving its implementation, interpretation, and acceptability. Our study thus provided further empirical evidence of the potential benefits of using sum scores in psychology and health sciences. Future studies should examine other practical or simulated cases to further evaluate the cost of simplicity and provide robust empirical evidence on this controversial issue.

## Supporting information

S1 Checklist *PLOS ONE* clinical studies checklist. (DOCX)

S2 Checklist STROBE statement—checklist of items that should be included in reports of observational studies. (DOCX)

S1 Table Items for self-reported alcohol use disorders and alcohol-related consequences. (DOCX)

S1 File Simplification algorithm. (DOCX)

S2 File R code. (R)

S1 Data Database. (TXT)
