# Supplementary material for: Evaluating the cost of simplicity in score building: An example from alcohol research
Source: PLoS One. 2023 Nov 27;18(11):e0294671. doi: 10.1371/journal.pone.0294671 (PMC10681198; doi:10.1371/journal.pone.0294671)
Supplement: S1 Table — (DOCX) [file pone.0294671.s003.docx]

**S1 Table. Items for self-reported alcohol use disorders and alcohol-related consequences**

| **Items** | | |
| --- | --- | --- |
| **Self-reported alcohol use disorder** | | |
|  | Q1 | Neglect role because of alcohol use |
|  | Q2 | Hazardous use of alcohol |
|  | Q3 | Social/interpersonal problems due to alcohol use |
|  | Q4 | Tolerance |
|  | Q5 | Withdrawal |
|  | Q6 | Use larger/longer than intended |
|  | Q7 | Unable to control/quit alcohol use |
|  | Q8 | Time spent getting/using/recovering alcohol |
|  | Q9 | Activities given up because of alcohol use |
|  | Q10 | Physical/psychological problems due to alcohol use |
|  | Q11 | Craving |
| **Self-reported alcohol-related consequences** | | |
|  | Q12 | I drank alcohol or took drugs or medicine in order to get over any of the bad secondary effects of drinking alcohol |
|  | Q13 | I had a mental blackout after drinking alcohol |
|  | Q14 | While drinking alcohol, I did something that I badly regretted later |
|  | Q15 | I had unplanned sex because I was drunk |
|  | Q16 | I had sex without a condom because I was drunk |
|  | Q17 | I had an accident or I got injured because I was drunk |
|  | Q18 | I came into a conflict with the police or with the authorities more than once because of my alcohol use |
|  | Q19 | I came into an argument or into a fight while drinking alcohol or straight after |
|  | Q20 | I damaged property, because I was drunk |
