# Supplementary material for: Evaluating the cost of simplicity in score building: An example from alcohol research
Source: PLoS One. 2023 Nov 27;18(11):e0294671. doi: 10.1371/journal.pone.0294671 (PMC10681198; doi:10.1371/journal.pone.0294671)
Supplement: S1 File — (DOCX) [file pone.0294671.s004.docx]

**S2 File. Simplification algorithm**

In what follows, we present an algorithm to “simplify” the coefficients of a “refined score”, yielding a “simple score”. One obvious way to do this is to replace the non-zero coefficients of a refined score by their signs, either “+1” or “-1”, yielding a simple score with only one possible digit. A slightly more sophisticated approach is to allow more than one possible digit (or integer) for the coefficients involved in a simple score. Consider a positive integer m, and the following 2m possible values for the (non-zero) coefficients in a simple score: -m, -(m-1), …, -2, -1, +1, +2, …, +(m-1), +m. Let c1, c2, …, cq the q (non-zero) coefficients of some refined score, and let cmax the maximum among the absolute values of the cj (for j=1,…,q). For technical reasons, we add an infinitely small positive value to cmax, so that cmax is strictly larger than the absolute values of all the cj. The corresponding simplified coefficients s1, s2, …, sq for j=1,…, q can then be defined as follows:

sj = INTEGER PART OF [sign(cj)*(1+m*abs(cj)/cmax)].

For example, with m=2 and cmax=2.5 (plus an infinitely small positive value), one gets:

sj=-2 if -2.5<=cj<-1.25

sj=-1 if -1.25<=cj<0

sj=+1 if 0<cj<=1.25

sj=+2 if 1.25<cj<=2.5.

This algorithm can be applied to simplify the coefficients of any refined score such as those obtained via the methods considered in this article.
