# Supplementary material for: Evaluating the cost of simplicity in score building: An example from alcohol research
Source: PLoS One. 2023 Nov 27;18(11):e0294671. doi: 10.1371/journal.pone.0294671 (PMC10681198; doi:10.1371/journal.pone.0294671)
Supplement: Attachment — Submitted filename: Response_reviewers_20230530.docx [file pone.0294671.s007.docx]

We thank the reviewers and editors for their interest in our study and for their meaningful comments which allowed us to improve our manuscript. We have responded to all comments and we hope that our manuscript is now suitable for publication in PLOS ONE.

**Journal Requirements:**

**1.** Please ensure that your manuscript meets PLOS ONE's style requirements, including those for file naming. The PLOS ONE style templates can be found at 
<https://journals.plos.org/plosone/s/file?id=wjVg/PLOSOne_formatting_sample_main_body.pdf> and 
<https://journals.plos.org/plosone/s/file?id=ba62/PLOSOne_formatting_sample_title_authors_affiliations.pdf>

**Response:** We updated the manuscript to follow PLOS ONE’s style requirements.

**2.** Thank you for stating the following financial disclosure: "This study was supported by Swiss National Research Foundation (no. 10001C_173418/1)."

Please state what role the funders took in the study.  If the funders had no role, please state: "The funders had no role in study design, data collection and analysis, decision to publish, or preparation of the manuscript."

If this statement is not correct you must amend it as needed. Please include this amended Role of Funder statement in your cover letter; we will change the online submission form on your behalf.

**Response:** The funder had no role in study design, data collection, and analysis. This sentence has been added to the cover letter.

**3.** We note that you have indicated that data from this study are available upon request. PLOS only allows data to be available upon request if there are legal or ethical restrictions on sharing data publicly. For more information on unacceptable data access restrictions, please see <http://journals.plos.org/plosone/s/data-availability#loc-unacceptable-data-access-restrictions>.

In your revised cover letter, please address the following prompts:

a) If there are ethical or legal restrictions on sharing a de-identified data set, please explain them in detail (e.g., data contain potentially sensitive information, data are owned by a third-party organization, etc.) and who has imposed them (e.g., an ethics committee). Please also provide contact information for a data access committee, ethics committee, or other institutional body to which data requests may be sent.

b) If there are no restrictions, please upload the minimal anonymized data set necessary to replicate your study findings as either Supporting Information files or to a stable, public repository and provide us with the relevant URLs, DOIs, or accession numbers. For a list of acceptable repositories, please see <http://journals.plos.org/plosone/s/data-availability#loc-recommended-repositories>.

We will update your Data Availability statement on your behalf to reflect the information you provide.

**Response:** Data and code are available as supplementary materials.

**4.** Your ethics statement should only appear in the Methods section of your manuscript. If your ethics statement is written in any section besides the Methods, please delete it from any other section.

**Response:** Our ethics statement only appears in the Methods section of the manuscript.

**5.** Please include captions for your Supporting Information files at the end of your manuscript, and update any in-text citations to match accordingly.

**Response:** The revised version of the manuscript includes captions for the Supporting Information files. In-text citations have been updated accordingly.

**Reviewer #1:**

**1.** It is recomended that not to use acronym in keywords. Author should add acronym and abbreviation in the keyword list.

**Response:** Thank you for the suggestion. We completed the list of keywords as follows:

**Keywords**: Area under the curve (AUC); Bootstrap; Least absolute shrinkage and selection operator (LASSO); Optimism; Overfitting; Prediction; Simplicity.

**2.** where is conclusion section?Author should add conclusion and clearly mention futurre work at the end of conclusion.

**Response:** We added the following conclusion to the manuscript:

**Conclusion**

To conclude, our example data demonstrated that simplifying a score to predict a binary outcome does not necessarily imply a major loss in statistical performance, while potentially improving its implementation, interpretation, and acceptability. Our study thus provided further empirical evidence of the potential benefits of using sum scores in psychology and health sciences. Future studies should examine other practical or simulated cases to further evaluate the cost of simplicity and provide robust empirical evidence on this controversial issue.

**3.** Author should add acronym and abbreviation in the keyword list.

**Response:** We added acronyms in the list of keywords (also see our response to comment #1).

**4.** Authos should discuss more paper in introducation as below

*An online survey and review about the awareness, coping style, and exercise behavior during the “COVID-19 pandemic situation” by implementing the cloud-based medical treatment technology system in China among the public

*A Prospective Observational Cohort of Clinical Outcomes in Medical Inpatients prescribed Pharmacological Thromboprophylaxis Using Different Clinical Risk Assessment Models(COMPT RAMs)

*Evaluating the validity evidence of an OSCE: Results from a new medical school

**Response:** Thank you for this suggestion. We agree that the introduction section was not clear enough. We have completely rewritten it. We first introduce the topic, then present the current knowledge and controversies about it, and then the gaps in knowledge that the paper aims to fill. We also completed the literature with recent advances in the field.

Relevant references added to the paper include:

2.	Edelsbrunner PA. A model and its fit lie in the eye of the beholder: Long live the sum score. Frontiers in Psychology. 2022;13. doi: 10.3389/fpsyg.2022.986767.

9.	McNeish D. Psychometric properties of sum scores and factor scores differ even when their correlation is 0.98: A response to Widaman and Revelle. Behavior Research Methods. 2022. doi: 10.3758/s13428-022-02016-x.

12.	Dawes RM. The robust beauty of improper linear models in decision making. American Psychologist. 1979;34:571-82. doi: 10.1037/0003-066X.34.7.571.

13.	Bobko P, Roth PL, Buster MA. The usefulness of unit weights in creating composite scores: A literature review, application to content validity, and meta-analysis. Organizational Research Methods. 2007;10(4):689-709. doi: 10.1177/1094428106294734.

16.	McNeish D. Limitations of the sum-and-alpha approach to measurement in behavioral research. Policy Insights from the Behavioral and Brain Sciences. 2022;9(2):196-203. doi: 10.1177/23727322221117144.

**5.** The scientific contribution and novelty should be explained more clearly. The research problem should be defined more clearly.

**Response:** We have restructured the Introduction section to better explain what was known and research gaps our study addressed (see response to your comment #4).

**6.** The authors should add some more background and motivation in the introduction.

**Response:** Please see our response to comment #4.

**7.** Add a paragaraph of contribution abd orgnization separtly.

**Response:** We are not sure to have understood this request. The authors’ contributions are described at the start of the paper (page 2). The organization of the paper is classical and follows PLOS One style requirements, including Introduction, Methods, Results, Discussion, and Conclusion sections. However, the Introduction has been re-organized, to better explain the context, goal, and strategy of our research, as explained above.

**8.** please give a proofread check to the paper.

**Response:** We proofread the paper and remove typos and grammatical errors. We apologize for these errors.

**Reviewer #2:**

**1.** The literature review is so poor that I couldn't establish related work. Authors need to redo this and clearly state their contributions linking them to literatures reviewed. Authors may wish to use the following to improve their literature review section.

DOI: 10.1371/journal.pone.0282812

DOI: 10.1007/s42235-022-00316-8

DOI: 10.1007/s11831-022-09850-4

DOI: 10.1371/journal.pone.0275346

**Response:** We agree that the introduction section was not clear enough. We have completely rewritten it. We first introduce the topic, then present the current knowledge and controversies about it, and then the gaps in knowledge that the paper aims to fill. We thank the reviewer for having proposed some references. However, the proposed references deal with optimization algorithms, which is not quite the purpose of our study, so they were not included in the revised version of the paper. On the other hand, we have included the following relevant references:

2.	Edelsbrunner PA. A model and its fit lie in the eye of the beholder: Long live the sum score. Frontiers in Psychology. 2022;13. doi: 10.3389/fpsyg.2022.986767.

9.	McNeish D. Psychometric properties of sum scores and factor scores differ even when their correlation is 0.98: A response to Widaman and Revelle. Behavior Research Methods. 2022. doi: 10.3758/s13428-022-02016-x.

12.	Dawes RM. The robust beauty of improper linear models in decision making. American Psychologist. 1979;34:571-82. doi: 10.1037/0003-066X.34.7.571.

13.	Bobko P, Roth PL, Buster MA. The usefulness of unit weights in creating composite scores: A literature review, application to content validity, and meta-analysis. Organizational Research Methods. 2007;10(4):689-709. doi: 10.1177/1094428106294734.

16.	McNeish D. Limitations of the sum-and-alpha approach to measurement in behavioral research. Policy Insights from the Behavioral and Brain Sciences. 2022;9(2):196-203. doi: 10.1177/23727322221117144.

**2.** The methodology does not permit a repeat of the research. Authors need to clearly articulate this.

**Response:** We made the statistical code available on SWISSUbase to allow replication. Please also see our response to comment #3 of the Journal Requirements. This is now mentioned at the end of the Methods section:

The statistical code and dataset are available on SWISSUbase (<https://www.swissubase.ch/en/researcher/my-studies/13494/16420/overview>).

**3.** the consequence of 1 and 2 above means the entire remaining sections need to be reworked.

**Response:** We reworked the whole manuscript according to comments #1 and #2.

**Reviewer #3:**

**1.** The introduction is lack of discussing some important relevant background. The literature review should survey the related studies, showing the latter’s weaknesses and gaps to highlight the contribution of the research. This section needs more consideration and the contribution should be clearly stated.

**Response:** Thank you for this suggestion. We agree that the introduction section was not clear enough. We have completely rewritten it. We first introduce the topic, then present the current knowledge and controversies about it, and then the gaps in knowledge that the paper aims to fill. We also completed the literature with recent advances in the field.

Relevant references added to the paper include:

2.	Edelsbrunner PA. A model and its fit lie in the eye of the beholder: Long live the sum score. Frontiers in Psychology. 2022;13. doi: 10.3389/fpsyg.2022.986767.

9.	McNeish D. Psychometric properties of sum scores and factor scores differ even when their correlation is 0.98: A response to Widaman and Revelle. Behavior Research Methods. 2022. doi: 10.3758/s13428-022-02016-x.

12.	Dawes RM. The robust beauty of improper linear models in decision making. American Psychologist. 1979;34:571-82. doi: 10.1037/0003-066X.34.7.571.

13.	Bobko P, Roth PL, Buster MA. The usefulness of unit weights in creating composite scores: A literature review, application to content validity, and meta-analysis. Organizational Research Methods. 2007;10(4):689-709. doi: 10.1177/1094428106294734.

16.	McNeish D. Limitations of the sum-and-alpha approach to measurement in behavioral research. Policy Insights from the Behavioral and Brain Sciences. 2022;9(2):196-203. doi: 10.1177/23727322221117144.

**2.** There should be a conclusion and future work section at the end of the manuscript.

**Response:** We added the following conclusion to the manuscript:

**Conclusion**

To conclude, our example data demonstrated that simplifying a score to predict a binary outcome does not necessarily imply a major loss in statistical performance, while potentially improving its implementation, interpretation, and acceptability. Our study thus provided further empirical evidence of the potential benefits of using sum scores in psychology and health sciences. Future studies should examine other practical or simulated cases to further evaluate the cost of simplicity and provide robust empirical evidence on this controversial issue.
