# Supplementary material for: Evaluating the cost of simplicity in score building: An example from alcohol research
Source: PLoS One. 2023 Nov 27;18(11):e0294671. doi: 10.1371/journal.pone.0294671 (PMC10681198; doi:10.1371/journal.pone.0294671)
Supplement: Attachment — Submitted filename: Response_reviewers_20230929.docx [file pone.0294671.s008.docx]

We thank the reviewer for his/er interest in our study and for the meaningful comments which allowed us to improve our manuscript. We have responded to all comments and we hope that our manuscript is now suitable for publication in PLOS ONE.

**Reviewer #4:**

**1.** Most of these modified models are slightly worse than the original one, whereas one is slightly better. The differences are not significant statistically. This observation is not surprising if one keeps in mind how logistic regression works: even if the weights obtained by the original logistic regression may seem to be unintuitive for the first sight, they are "optimal" in the sense that logistic regression aims to minimize a particular loss function (cross-entropy), therefore slight modifications of these weights are not expected to be better in general. On the other hand, due to the robustness of logistic regression, slight modifications of the weights are not expected to lead to a catastrophic decrease of performance either.

**Response:** Like the reviewer, we are personally not too surprised to find that statistical performance is not much impaired when the coefficients of our logistic regression are rounded or simplified to some extent. But some researchers will certainly be, as witnessed by the controversy on simple scores reported in our introduction. The aim of our article is precisely to illustrate (or confirm, for those who already suspected it) via a real example that such a procedure can be applied with only a slight loss (and sometimes even with a gain) in statistical performance. This is good news, since simple scores have other advantages, such as ease of implementation, interpretation, or acceptability, as we wrote in our article. Although we are aware that our article does not constitute a breakthrough, we think it is useful to give simple scores some publicity, and to encourage, or at least not penalize, researchers who would like to use and develop them.

**2.** The observations reported in the manuscript are only based on a single dataset only.

**Response:** We agree this is a study limitation. It is now explicitly mentioned as a limitation in the discussion section.

These recommendations are based on a single, albeit real, data example, which constitutes a study limitation.

**3.** Additionally, some details are questionable. For example, as for the persons with low score of alcohol use, a random selection has been made, and 137 persons have been selected. I wonder why exactly 137 patients were selected, why not 193 which would match the number of persons with high score? The authors write that "the final sample size was 233", however, 137+193=230.

**Response:** The final sample size we wanted to reach in the main study was n=86 in each group (total n=172). However, this corresponds to the number of participants needed for the gold standard (diagnosis of AUD). At the time of participants’ selection, we only had the AUDIT score, and we were thus likely to have false positives and false negatives in both groups.

As the number of false positives was likely to be high in the high AUDIT score group, we increased the number of participants we invited to participate in this group. In total, we invited 330 participants (137+193), of which 230 agreed to participate. We finally got n=78 with a diagnosis of AUD. This suggests a high proportion of false positives according to the AUDIT (see a comment we wrote about it: Baggio, S., & Iglesias, K. (2020). On the limitations of the alcohol use disorders identification test (AUDIT). *Drug and Alcohol Dependence,* 206, 107662).

We removed the sentence on the number of participants invited in each stratum, as it was confusing. Instead, we report response rates separately for the two strata.

Participants were then selected using a stratified sampling strategy: those with a high AUDIT score (≥13) and those with a low score (<13) [21]. The final sample size was 233 (total response rate=70.6%, 68.9% in the low-strata group and 72.0% in the high-strata group).

**4.** Last, but not least, I don't understand why the Authors used Steyerberg's procedure for correction of AUC values instead of evaluating the model on a test set which is disjoint from the training data.

**Response:** The paper we cite from Steyerberg et al, which has been cited more than 1800 times in the literature, compares several methods for correcting optimism in the context of logistic regression, and recommends bootstrapping over a split-sample procedure, which was the reason for our choice. But whatever the method used, it is already a good thing to try to correct for optimism, as this practice is far from always being adopted by researchers.

In addition, the sample size was relatively modest. This is now mentioned in the study’s limitations.

In addition, the sample size was relatively small, which did not allow splitting the dataset into train and test sets.
