# Supplementary material for: Evaluating the cost of simplicity in score building: An example from alcohol research
Source: PLoS One. 2023 Nov 27;18(11):e0294671. doi: 10.1371/journal.pone.0294671 (PMC10681198; doi:10.1371/journal.pone.0294671)
Supplement: Peer review history

# Peer review: Decision Letter 0

**DOI:** 10.1371/journal.pone.0294671.r001

**Authors:** Abdullah Almuhaimeed

10 Apr 2023

PONE-D-23-07943Evaluating the cost of simplicity in score building: An example from alcohol researchPLOS ONE

Dear Dr. Baggio,

Thank you for submitting your manuscript to PLOS ONE. After careful consideration, we feel that it has merit but does not fully meet PLOS ONE’s publication criteria as it currently stands. Therefore, we invite you to submit a revised version of the manuscript that addresses the points raised during the review process.

Please submit your revised manuscript by May 25 2023 11:59PM. If you will need more time than this to complete your revisions, please reply to this message or contact the journal office at plosone@plos.org. When you're ready to submit your revision, log on to https://www.editorialmanager.com/pone/ and select the 'Submissions Needing Revision' folder to locate your manuscript file.

Please include the following items when submitting your revised manuscript:

- A rebuttal letter that responds to each point raised by the academic editor and reviewer(s). You should upload this letter as a separate file labeled 'Response to Reviewers'.
- A marked-up copy of your manuscript that highlights changes made to the original version. You should upload this as a separate file labeled 'Revised Manuscript with Track Changes'.
- An unmarked version of your revised paper without tracked changes. You should upload this as a separate file labeled 'Manuscript'.

If you would like to make changes to your financial disclosure, please include your updated statement in your cover letter. Guidelines for resubmitting your figure files are available below the reviewer comments at the end of this letter.

If applicable, we recommend that you deposit your laboratory protocols in protocols.io to enhance the reproducibility of your results. Protocols.io assigns your protocol its own identifier (DOI) so that it can be cited independently in the future. For instructions see: https://journals.plos.org/plosone/s/submission-guidelines#loc-laboratory-protocols. Additionally, PLOS ONE offers an option for publishing peer-reviewed Lab Protocol articles, which describe protocols hosted on protocols.io. Read more information on sharing protocols at https://plos.org/protocols?utm_medium=editorial-email&utm_source=authorletters&utm_campaign=protocols.

We look forward to receiving your revised manuscript.

Kind regards,

Abdullah Almuhaimeed

Academic Editor

PLOS ONE

Journal Requirements:

When submitting your revision, we need you to address these additional requirements.

1. Please ensure that your manuscript meets PLOS ONE's style requirements, including those for file naming. The PLOS ONE style templates can be found at 

https://journals.plos.org/plosone/s/file?id=wjVg/PLOSOne_formatting_sample_main_body.pdf and 

https://journals.plos.org/plosone/s/file?id=ba62/PLOSOne_formatting_sample_title_authors_affiliations.pdf

2. Thank you for stating the following financial disclosure: 

   "This study was supported by Swiss National Research Foundation (no. 10001C_173418/1)."

Please state what role the funders took in the study.  If the funders had no role, please state: "The funders had no role in study design, data collection and analysis, decision to publish, or preparation of the manuscript." 

If this statement is not correct you must amend it as needed. 

Please include this amended Role of Funder statement in your cover letter; we will change the online submission form on your behalf.

3. We note that you have indicated that data from this study are available upon request. PLOS only allows data to be available upon request if there are legal or ethical restrictions on sharing data publicly. For more information on unacceptable data access restrictions, please see http://journals.plos.org/plosone/s/data-availability#loc-unacceptable-data-access-restrictions. 

In your revised cover letter, please address the following prompts:

a) If there are ethical or legal restrictions on sharing a de-identified data set, please explain them in detail (e.g., data contain potentially sensitive information, data are owned by a third-party organization, etc.) and who has imposed them (e.g., an ethics committee). Please also provide contact information for a data access committee, ethics committee, or other institutional body to which data requests may be sent.

b) If there are no restrictions, please upload the minimal anonymized data set necessary to replicate your study findings as either Supporting Information files or to a stable, public repository and provide us with the relevant URLs, DOIs, or accession numbers. For a list of acceptable repositories, please see http://journals.plos.org/plosone/s/data-availability#loc-recommended-repositories.

We will update your Data Availability statement on your behalf to reflect the information you provide.

4. Your ethics statement should only appear in the Methods section of your manuscript. If your ethics statement is written in any section besides the Methods, please delete it from any other section. 

5. Please include captions for your Supporting Information files at the end of your manuscript, and update any in-text citations to match accordingly. Please see our Supporting Information guidelines for more information: http://journals.plos.org/plosone/s/supporting-information. 

Additional Editor Comments:

Dear Stéphanie Baggio,

Thank you for submitting your work to the Plos One journal. Your work suffers from some issues that mentioned by the reviewers. Please revise your work and re-submit it again to the journal.

Thank you.

[Note: HTML markup is below. Please do not edit.]

Reviewers' comments:

Reviewer's Responses to Questions

**Comments to the Author**

1. Is the manuscript technically sound, and do the data support the conclusions?

The manuscript must describe a technically sound piece of scientific research with data that supports the conclusions. Experiments must have been conducted rigorously, with appropriate controls, replication, and sample sizes. The conclusions must be drawn appropriately based on the data presented. 

Reviewer #1: No

Reviewer #2: Partly

Reviewer #3: Partly

**********

2. Has the statistical analysis been performed appropriately and rigorously? 

Reviewer #1: N/A

Reviewer #2: Yes

Reviewer #3: Yes

**********

3. Have the authors made all data underlying the findings in their manuscript fully available?

The PLOS Data policy requires authors to make all data underlying the findings described in their manuscript fully available without restriction, with rare exception (please refer to the Data Availability Statement in the manuscript PDF file). The data should be provided as part of the manuscript or its supporting information, or deposited to a public repository. For example, in addition to summary statistics, the data points behind means, medians and variance measures should be available. If there are restrictions on publicly sharing data—e.g. participant privacy or use of data from a third party—those must be specified.

Reviewer #1: Yes

Reviewer #2: Yes

Reviewer #3: Yes

**********

4. Is the manuscript presented in an intelligible fashion and written in standard English?

PLOS ONE does not copyedit accepted manuscripts, so the language in submitted articles must be clear, correct, and unambiguous. Any typographical or grammatical errors should be corrected at revision, so please note any specific errors here.

Reviewer #1: Yes

Reviewer #2: No

Reviewer #3: No

**********

5. Review Comments to the Author

Please use the space provided to explain your answers to the questions above. You may also include additional comments for the author, including concerns about dual publication, research ethics, or publication ethics. (Please upload your review as an attachment if it exceeds 20,000 characters)

Reviewer #1: It is recomended that not to use acronym in keywords. Author should add acronym and abbreviation in the keyword list.

where is conclusion section?Author should add conclusion and clearly mention futurre work at the end of conclusion.

Author should add acronym and abbreviation in the keyword list.

Authos should discuss more paper in introducation as below

*An online survey and review about the awareness, coping style, and exercise behavior during the “COVID-19 pandemic situation” by implementing the cloud-based medical treatment technology system in China among the public

*A Prospective Observational Cohort of Clinical Outcomes in Medical Inpatients prescribed Pharmacological Thromboprophylaxis Using Different Clinical Risk Assessment Models(COMPT RAMs)

*Evaluating the validity evidence of an OSCE: Results from a new medical school

The scientific contribution and novelty should be explained more clearly. The research problem should be defined more clearly.

The authors should add some more background and motivation in the introduction.

Add a paragaraph of contribution abd orgnization separtly.

please give a proofread check to the paper.

Reviewer #2: The article though interesting, the following are not clear to me.

1. The literature review is so poor that I couldn't establish related work. Authors need to redo this and clearly state their contributions linking them to literatures reviewed. Authors may wish to use the following to improve their literature review section.

DOI: 10.1371/journal.pone.0282812

DOI: 10.1007/s42235-022-00316-8

DOI: 10.1007/s11831-022-09850-4

DOI: 10.1371/journal.pone.0275346

2. The methodology does not permit a repeat of the research. Authors need to clearly articulate this.

3. the consequence of 1 and 2 above means the entire remaining sections need to be reworked.

Reviewer #3: The introduction is lack of discussing some important relevant background.

The literature review should survey the related studies, showing the latter’s weaknesses and gaps to highlight the contribution of the research. This section needs more consideration and the contribution should be clearly stated.

There should be a conclusion and future work section at the end of the manuscript.

**********

6. PLOS authors have the option to publish the peer review history of their article (what does this mean?). If published, this will include your full peer review and any attached files.

If you choose “no”, your identity will remain anonymous but your review may still be made public.

**Do you want your identity to be public for this peer review?** For information about this choice, including consent withdrawal, please see our Privacy Policy.

Reviewer #1: No

Reviewer #2: No

Reviewer #3: No

**********

[NOTE: If reviewer comments were submitted as an attachment file, they will be attached to this email and accessible via the submission site. Please log into your account, locate the manuscript record, and check for the action link "View Attachments". If this link does not appear, there are no attachment files.]

While revising your submission, please upload your figure files to the Preflight Analysis and Conversion Engine (PACE) digital diagnostic tool, https://pacev2.apexcovantage.com/. PACE helps ensure that figures meet PLOS requirements. To use PACE, you must first register as a user. Registration is free. Then, login and navigate to the UPLOAD tab, where you will find detailed instructions on how to use the tool. If you encounter any issues or have any questions when using PACE, please email PLOS at figures@plos.org. Please note that Supporting Information files do not need this step.

# Author comment: Author response to Decision Letter 0

**DOI:** 10.1371/journal.pone.0294671.r002

30 May 2023

We thank the reviewers and editors for their interest in our study and for their meaningful comments which allowed us to improve our manuscript. We have responded to all comments and we hope that our manuscript is now suitable for publication in PLOS ONE.

Journal Requirements:

1. Please ensure that your manuscript meets PLOS ONE's style requirements, including those for file naming. The PLOS ONE style templates can be found at 

https://journals.plos.org/plosone/s/file?id=wjVg/PLOSOne_formatting_sample_main_body.pdf and 

https://journals.plos.org/plosone/s/file?id=ba62/PLOSOne_formatting_sample_title_authors_affiliations.pdf

Response: We updated the manuscript to follow PLOS ONE’s style requirements.

2. Thank you for stating the following financial disclosure: "This study was supported by Swiss National Research Foundation (no. 10001C_173418/1)."

Please state what role the funders took in the study. If the funders had no role, please state: "The funders had no role in study design, data collection and analysis, decision to publish, or preparation of the manuscript." 

If this statement is not correct you must amend it as needed. Please include this amended Role of Funder statement in your cover letter; we will change the online submission form on your behalf.

Response: The funder had no role in study design, data collection, and analysis. This sentence has been added to the cover letter.

3. We note that you have indicated that data from this study are available upon request. PLOS only allows data to be available upon request if there are legal or ethical restrictions on sharing data publicly. For more information on unacceptable data access restrictions, please see http://journals.plos.org/plosone/s/data-availability#loc-unacceptable-data-access-restrictions. 

In your revised cover letter, please address the following prompts:

a) If there are ethical or legal restrictions on sharing a de-identified data set, please explain them in detail (e.g., data contain potentially sensitive information, data are owned by a third-party organization, etc.) and who has imposed them (e.g., an ethics committee). Please also provide contact information for a data access committee, ethics committee, or other institutional body to which data requests may be sent.

b) If there are no restrictions, please upload the minimal anonymized data set necessary to replicate your study findings as either Supporting Information files or to a stable, public repository and provide us with the relevant URLs, DOIs, or accession numbers. For a list of acceptable repositories, please see http://journals.plos.org/plosone/s/data-availability#loc-recommended-repositories.

We will update your Data Availability statement on your behalf to reflect the information you provide.

Response: Data and code are available as supplementary materials. 

4. Your ethics statement should only appear in the Methods section of your manuscript. If your ethics statement is written in any section besides the Methods, please delete it from any other section. 

Response: Our ethics statement only appears in the Methods section of the manuscript.

5. Please include captions for your Supporting Information files at the end of your manuscript, and update any in-text citations to match accordingly. 

Response: The revised version of the manuscript includes captions for the Supporting Information files. In-text citations have been updated accordingly.

Reviewer #1: 

1. It is recomended that not to use acronym in keywords. Author should add acronym and abbreviation in the keyword list.

Response: Thank you for the suggestion. We completed the list of keywords as follows:

Keywords: Area under the curve (AUC); Bootstrap; Least absolute shrinkage and selection operator (LASSO); Optimism; Overfitting; Prediction; Simplicity.

2. where is conclusion section?Author should add conclusion and clearly mention futurre work at the end of conclusion.

Response: We added the following conclusion to the manuscript:

Conclusion

To conclude, our example data demonstrated that simplifying a score to predict a binary outcome does not necessarily imply a major loss in statistical performance, while potentially improving its implementation, interpretation, and acceptability. Our study thus provided further empirical evidence of the potential benefits of using sum scores in psychology and health sciences. Future studies should examine other practical or simulated cases to further evaluate the cost of simplicity and provide robust empirical evidence on this controversial issue.

3. Author should add acronym and abbreviation in the keyword list.

Response: We added acronyms in the list of keywords (also see our response to comment #1).

4. Authos should discuss more paper in introducation as below

*An online survey and review about the awareness, coping style, and exercise behavior during the “COVID-19 pandemic situation” by implementing the cloud-based medical treatment technology system in China among the public

*A Prospective Observational Cohort of Clinical Outcomes in Medical Inpatients prescribed Pharmacological Thromboprophylaxis Using Different Clinical Risk Assessment Models(COMPT RAMs)

*Evaluating the validity evidence of an OSCE: Results from a new medical school

Response: Thank you for this suggestion. We agree that the introduction section was not clear enough. We have completely rewritten it. We first introduce the topic, then present the current knowledge and controversies about it, and then the gaps in knowledge that the paper aims to fill. We also completed the literature with recent advances in the field.

Relevant references added to the paper include:

2. Edelsbrunner PA. A model and its fit lie in the eye of the beholder: Long live the sum score. Frontiers in Psychology. 2022;13. doi: 10.3389/fpsyg.2022.986767.

9. McNeish D. Psychometric properties of sum scores and factor scores differ even when their correlation is 0.98: A response to Widaman and Revelle. Behavior Research Methods. 2022. doi: 10.3758/s13428-022-02016-x.

12. Dawes RM. The robust beauty of improper linear models in decision making. American Psychologist. 1979;34:571-82. doi: 10.1037/0003-066X.34.7.571.

13. Bobko P, Roth PL, Buster MA. The usefulness of unit weights in creating composite scores: A literature review, application to content validity, and meta-analysis. Organizational Research Methods. 2007;10(4):689-709. doi: 10.1177/1094428106294734.

16. McNeish D. Limitations of the sum-and-alpha approach to measurement in behavioral research. Policy Insights from the Behavioral and Brain Sciences. 2022;9(2):196-203. doi: 10.1177/23727322221117144.

5. The scientific contribution and novelty should be explained more clearly. The research problem should be defined more clearly.

Response: We have restructured the Introduction section to better explain what was known and research gaps our study addressed (see response to your comment #4). 

6. The authors should add some more background and motivation in the introduction.

Response: Please see our response to comment #4.

7. Add a paragaraph of contribution abd orgnization separtly.

Response: We are not sure to have understood this request. The authors’ contributions are described at the start of the paper (page 2). The organization of the paper is classical and follows PLOS One style requirements, including Introduction, Methods, Results, Discussion, and Conclusion sections. However, the Introduction has been re-organized, to better explain the context, goal, and strategy of our research, as explained above. 

8. please give a proofread check to the paper.

Response: We proofread the paper and remove typos and grammatical errors. We apologize for these errors.

Reviewer #2: 

1. The literature review is so poor that I couldn't establish related work. Authors need to redo this and clearly state their contributions linking them to literatures reviewed. Authors may wish to use the following to improve their literature review section.

DOI: 10.1371/journal.pone.0282812

DOI: 10.1007/s42235-022-00316-8

DOI: 10.1007/s11831-022-09850-4

DOI: 10.1371/journal.pone.0275346

Response: We agree that the introduction section was not clear enough. We have completely rewritten it. We first introduce the topic, then present the current knowledge and controversies about it, and then the gaps in knowledge that the paper aims to fill. We thank the reviewer for having proposed some references. However, the proposed references deal with optimization algorithms, which is not quite the purpose of our study, so they were not included in the revised version of the paper. On the other hand, we have included the following relevant references:

2. Edelsbrunner PA. A model and its fit lie in the eye of the beholder: Long live the sum score. Frontiers in Psychology. 2022;13. doi: 10.3389/fpsyg.2022.986767.

9. McNeish D. Psychometric properties of sum scores and factor scores differ even when their correlation is 0.98: A response to Widaman and Revelle. Behavior Research Methods. 2022. doi: 10.3758/s13428-022-02016-x.

12. Dawes RM. The robust beauty of improper linear models in decision making. American Psychologist. 1979;34:571-82. doi: 10.1037/0003-066X.34.7.571.

13. Bobko P, Roth PL, Buster MA. The usefulness of unit weights in creating composite scores: A literature review, application to content validity, and meta-analysis. Organizational Research Methods. 2007;10(4):689-709. doi: 10.1177/1094428106294734.

16. McNeish D. Limitations of the sum-and-alpha approach to measurement in behavioral research. Policy Insights from the Behavioral and Brain Sciences. 2022;9(2):196-203. doi: 10.1177/23727322221117144.

2. The methodology does not permit a repeat of the research. Authors need to clearly articulate this.

Response: We made the statistical code available on SWISSUbase to allow replication. Please also see our response to comment #3 of the Journal Requirements. This is now mentioned at the end of the Methods section:

The statistical code and dataset are available on SWISSUbase (https://www.swissubase.ch/en/researcher/my-studies/13494/16420/overview).

3. the consequence of 1 and 2 above means the entire remaining sections need to be reworked.

Response: We reworked the whole manuscript according to comments #1 and #2.

Reviewer #3: 

1. The introduction is lack of discussing some important relevant background. The literature review should survey the related studies, showing the latter’s weaknesses and gaps to highlight the contribution of the research. This section needs more consideration and the contribution should be clearly stated.

Response: Thank you for this suggestion. We agree that the introduction section was not clear enough. We have completely rewritten it. We first introduce the topic, then present the current knowledge and controversies about it, and then the gaps in knowledge that the paper aims to fill. We also completed the literature with recent advances in the field.

Relevant references added to the paper include:

2. Edelsbrunner PA. A model and its fit lie in the eye of the beholder: Long live the sum score. Frontiers in Psychology. 2022;13. doi: 10.3389/fpsyg.2022.986767.

9. McNeish D. Psychometric properties of sum scores and factor scores differ even when their correlation is 0.98: A response to Widaman and Revelle. Behavior Research Methods. 2022. doi: 10.3758/s13428-022-02016-x.

12. Dawes RM. The robust beauty of improper linear models in decision making. American Psychologist. 1979;34:571-82. doi: 10.1037/0003-066X.34.7.571.

13. Bobko P, Roth PL, Buster MA. The usefulness of unit weights in creating composite scores: A literature review, application to content validity, and meta-analysis. Organizational Research Methods. 2007;10(4):689-709. doi: 10.1177/1094428106294734.

16. McNeish D. Limitations of the sum-and-alpha approach to measurement in behavioral research. Policy Insights from the Behavioral and Brain Sciences. 2022;9(2):196-203. doi: 10.1177/23727322221117144.

2. There should be a conclusion and future work section at the end of the manuscript.

Response: We added the following conclusion to the manuscript:

Conclusion

To conclude, our example data demonstrated that simplifying a score to predict a binary outcome does not necessarily imply a major loss in statistical performance, while potentially improving its implementation, interpretation, and acceptability. Our study thus provided further empirical evidence of the potential benefits of using sum scores in psychology and health sciences. Future studies should examine other practical or simulated cases to further evaluate the cost of simplicity and provide robust empirical evidence on this controversial issue.

Attachment Submitted filename: Response_reviewers_20230530.docx Click here for additional data file.

# Peer review: Decision Letter 1

**DOI:** 10.1371/journal.pone.0294671.r003

**Authors:** Sathishkumar Veerappampalayam Easwaramoorthy

26 Sep 2023

PONE-D-23-07943R1Evaluating the cost of simplicity in score building: An example from alcohol researchPLOS ONE

Dear Dr. Baggio,

Thank you for submitting your manuscript to PLOS ONE. After careful consideration, we feel that it has merit but does not fully meet PLOS ONE’s publication criteria as it currently stands. Therefore, we invite you to submit a revised version of the manuscript that addresses the points raised during the review process.

Please submit your revised manuscript by Nov 10 2023 11:59PM. If you will need more time than this to complete your revisions, please reply to this message or contact the journal office at plosone@plos.org. When you're ready to submit your revision, log on to https://www.editorialmanager.com/pone/ and select the 'Submissions Needing Revision' folder to locate your manuscript file.

Please include the following items when submitting your revised manuscript:

- A rebuttal letter that responds to each point raised by the academic editor and reviewer(s). You should upload this letter as a separate file labeled 'Response to Reviewers'.
- A marked-up copy of your manuscript that highlights changes made to the original version. You should upload this as a separate file labeled 'Revised Manuscript with Track Changes'.
- An unmarked version of your revised paper without tracked changes. You should upload this as a separate file labeled 'Manuscript'.

If you would like to make changes to your financial disclosure, please include your updated statement in your cover letter. Guidelines for resubmitting your figure files are available below the reviewer comments at the end of this letter.

If applicable, we recommend that you deposit your laboratory protocols in protocols.io to enhance the reproducibility of your results. Protocols.io assigns your protocol its own identifier (DOI) so that it can be cited independently in the future. For instructions see: https://journals.plos.org/plosone/s/submission-guidelines#loc-laboratory-protocols. Additionally, PLOS ONE offers an option for publishing peer-reviewed Lab Protocol articles, which describe protocols hosted on protocols.io. Read more information on sharing protocols at https://plos.org/protocols?utm_medium=editorial-email&utm_source=authorletters&utm_campaign=protocols.

We look forward to receiving your revised manuscript.

Kind regards,

Sathishkumar Veerappampalayam Easwaramoorthy

Academic Editor

PLOS ONE

[Note: HTML markup is below. Please do not edit.]

Reviewers' comments:

Reviewer's Responses to Questions

**Comments to the Author**

1. If the authors have adequately addressed your comments raised in a previous round of review and you feel that this manuscript is now acceptable for publication, you may indicate that here to bypass the “Comments to the Author” section, enter your conflict of interest statement in the “Confidential to Editor” section, and submit your "Accept" recommendation.

Reviewer #2: All comments have been addressed

Reviewer #4: (No Response)

Reviewer #5: All comments have been addressed

**********

2. Is the manuscript technically sound, and do the data support the conclusions?

The manuscript must describe a technically sound piece of scientific research with data that supports the conclusions. Experiments must have been conducted rigorously, with appropriate controls, replication, and sample sizes. The conclusions must be drawn appropriately based on the data presented. 

Reviewer #2: (No Response)

Reviewer #4: No

Reviewer #5: Yes

**********

3. Has the statistical analysis been performed appropriately and rigorously? 

Reviewer #2: (No Response)

Reviewer #4: No

Reviewer #5: Yes

**********

4. Have the authors made all data underlying the findings in their manuscript fully available?

The PLOS Data policy requires authors to make all data underlying the findings described in their manuscript fully available without restriction, with rare exception (please refer to the Data Availability Statement in the manuscript PDF file). The data should be provided as part of the manuscript or its supporting information, or deposited to a public repository. For example, in addition to summary statistics, the data points behind means, medians and variance measures should be available. If there are restrictions on publicly sharing data—e.g. participant privacy or use of data from a third party—those must be specified.

Reviewer #2: (No Response)

Reviewer #4: Yes

Reviewer #5: Yes

**********

5. Is the manuscript presented in an intelligible fashion and written in standard English?

PLOS ONE does not copyedit accepted manuscripts, so the language in submitted articles must be clear, correct, and unambiguous. Any typographical or grammatical errors should be corrected at revision, so please note any specific errors here.

Reviewer #2: (No Response)

Reviewer #4: Yes

Reviewer #5: Yes

**********

6. Review Comments to the Author

Please use the space provided to explain your answers to the questions above. You may also include additional comments for the author, including concerns about dual publication, research ethics, or publication ethics. (Please upload your review as an attachment if it exceeds 20,000 characters)

Reviewer #2: (No Response)

Reviewer #4: The manuscript considers the problem of alcohol use disorder. Based on the answers of a survey, two groups of persons are defined: with and without alcohol use disorder. Thus, a binary decision problem is defined which is solved by logistic regression, a standard statistical approach. Subsequently, the Authors evaluate the models resulting from slight modification of the coefficients obtained by logistic regression (such as rounding the coefficients to 3, 2 and 1 digit(s), setting negative coefficients to zero because based on domain knowledge it is known that all the variables correlate with the outcome positively). Most of these modified models are slightly worse than the original one, whereas one is slightly better. The differences are not significant statistically. This observation is not surprising if one keeps in mind how logistic regression works: even if the weights obtained by the original logistic regression may seem to be unintuitive for the first sight, they are "optimal" in the sense that logistic regression aims to minimize a particular loss function (cross-entropy), therefore slight modifications of these weights are not expected to be better in general. On the other hand, due to the robustness of logistic regression, slight modifications of the weights are not expected to lead to a catastrophic decrease of performance either.

The observations reported in the manuscript are only based on a single dataset only.

Additionally, some details are questionable. For example, as for the persons with low score of alcohol use, a random selection has been made, and 137 persons have been selected. I wonder why exactly 137 patients were selected, why not 193 which would match the number of persons with high score? The authors write that "the final sample size was 233", however, 137+193=230. Last, but not least, I don't understand why the Authors used Steyerberg's procedure for correction of AUC values instead of evaluating the model on a test set which is disjoint from the training data.

Reviewer #5: The initial manuscript was reviewed by three independent reviewers other than myself. However, the authors have adequately answered the comments raised by the reviewers, and I believe the article can be published.

**********

7. PLOS authors have the option to publish the peer review history of their article (what does this mean?). If published, this will include your full peer review and any attached files.

If you choose “no”, your identity will remain anonymous but your review may still be made public.

**Do you want your identity to be public for this peer review?** For information about this choice, including consent withdrawal, please see our Privacy Policy.

Reviewer #2: No

Reviewer #4: No

Reviewer #5: No

**********

[NOTE: If reviewer comments were submitted as an attachment file, they will be attached to this email and accessible via the submission site. Please log into your account, locate the manuscript record, and check for the action link "View Attachments". If this link does not appear, there are no attachment files.]

While revising your submission, please upload your figure files to the Preflight Analysis and Conversion Engine (PACE) digital diagnostic tool, https://pacev2.apexcovantage.com/. PACE helps ensure that figures meet PLOS requirements. To use PACE, you must first register as a user. Registration is free. Then, login and navigate to the UPLOAD tab, where you will find detailed instructions on how to use the tool. If you encounter any issues or have any questions when using PACE, please email PLOS at figures@plos.org. Please note that Supporting Information files do not need this step.

# Author comment: Author response to Decision Letter 1

**DOI:** 10.1371/journal.pone.0294671.r004

2 Oct 2023

We thank the reviewer for his/er interest in our study and for the meaningful comments which allowed us to improve our manuscript. We have responded to all comments and we hope that our manuscript is now suitable for publication in PLOS ONE.

Reviewer #4: 

1. Most of these modified models are slightly worse than the original one, whereas one is slightly better. The differences are not significant statistically. This observation is not surprising if one keeps in mind how logistic regression works: even if the weights obtained by the original logistic regression may seem to be unintuitive for the first sight, they are "optimal" in the sense that logistic regression aims to minimize a particular loss function (cross-entropy), therefore slight modifications of these weights are not expected to be better in general. On the other hand, due to the robustness of logistic regression, slight modifications of the weights are not expected to lead to a catastrophic decrease of performance either.

Response: Like the reviewer, we are personally not too surprised to find that statistical performance is not much impaired when the coefficients of our logistic regression are rounded or simplified to some extent. But some researchers will certainly be, as witnessed by the controversy on simple scores reported in our introduction. The aim of our article is precisely to illustrate (or confirm, for those who already suspected it) via a real example that such a procedure can be applied with only a slight loss (and sometimes even with a gain) in statistical performance. This is good news, since simple scores have other advantages, such as ease of implementation, interpretation, or acceptability, as we wrote in our article. Although we are aware that our article does not constitute a breakthrough, we think it is useful to give simple scores some publicity, and to encourage, or at least not penalize, researchers who would like to use and develop them.

2. The observations reported in the manuscript are only based on a single dataset only.

Response: We agree this is a study limitation. It is now explicitly mentioned as a limitation in the discussion section.

These recommendations are based on a single, albeit real, data example, which constitutes a study limitation.

3. Additionally, some details are questionable. For example, as for the persons with low score of alcohol use, a random selection has been made, and 137 persons have been selected. I wonder why exactly 137 patients were selected, why not 193 which would match the number of persons with high score? The authors write that "the final sample size was 233", however, 137+193=230. 

Response: The final sample size we wanted to reach in the main study was n=86 in each group (total n=172). However, this corresponds to the number of participants needed for the gold standard (diagnosis of AUD). At the time of participants’ selection, we only had the AUDIT score, and we were thus likely to have false positives and false negatives in both groups. 

As the number of false positives was likely to be high in the high AUDIT score group, we increased the number of participants we invited to participate in this group. In total, we invited 330 participants (137+193), of which 230 agreed to participate. We finally got n=78 with a diagnosis of AUD. This suggests a high proportion of false positives according to the AUDIT (see a comment we wrote about it: Baggio, S., & Iglesias, K. (2020). On the limitations of the alcohol use disorders identification test (AUDIT). Drug and Alcohol Dependence, 206, 107662).

We removed the sentence on the number of participants invited in each stratum, as it was confusing. Instead, we report response rates separately for the two strata.

Participants were then selected using a stratified sampling strategy: those with a high AUDIT score (≥13) and those with a low score (<13) [21]. The final sample size was 233 (total response rate=70.6%, 68.9% in the low-strata group and 72.0% in the high-strata group).

4. Last, but not least, I don't understand why the Authors used Steyerberg's procedure for correction of AUC values instead of evaluating the model on a test set which is disjoint from the training data.

Response: The paper we cite from Steyerberg et al, which has been cited more than 1800 times in the literature, compares several methods for correcting optimism in the context of logistic regression, and recommends bootstrapping over a split-sample procedure, which was the reason for our choice. But whatever the method used, it is already a good thing to try to correct for optimism, as this practice is far from always being adopted by researchers.

In addition, the sample size was relatively modest. This is now mentioned in the study’s limitations.

In addition, the sample size was relatively small, which did not allow splitting the dataset into train and test sets.

Attachment Submitted filename: Response_reviewers_20230929.docx Click here for additional data file.

# Peer review: Decision Letter 2

**DOI:** 10.1371/journal.pone.0294671.r005

**Authors:** Sathishkumar Veerappampalayam Easwaramoorthy

7 Nov 2023

Evaluating the cost of simplicity in score building: An example from alcohol research

PONE-D-23-07943R2

Dear Dr. Baggio,

We’re pleased to inform you that your manuscript has been judged scientifically suitable for publication and will be formally accepted for publication once it meets all outstanding technical requirements.

Within one week, you’ll receive an e-mail detailing the required amendments. When these have been addressed, you’ll receive a formal acceptance letter and your manuscript will be scheduled for publication.

An invoice for payment will follow shortly after the formal acceptance. To ensure an efficient process, please log into Editorial Manager at http://www.editorialmanager.com/pone/, click the 'Update My Information' link at the top of the page, and double check that your user information is up-to-date. If you have any billing related questions, please contact our Author Billing department directly at authorbilling@plos.org.

If your institution or institutions have a press office, please notify them about your upcoming paper to help maximize its impact. If they’ll be preparing press materials, please inform our press team as soon as possible -- no later than 48 hours after receiving the formal acceptance. Your manuscript will remain under strict press embargo until 2 pm Eastern Time on the date of publication. For more information, please contact onepress@plos.org.

Kind regards,

Sathishkumar Veerappampalayam Easwaramoorthy

Academic Editor

PLOS ONE

Additional Editor Comments (optional):

Reviewers' comments:

Reviewer's Responses to Questions

**Comments to the Author**

1. If the authors have adequately addressed your comments raised in a previous round of review and you feel that this manuscript is now acceptable for publication, you may indicate that here to bypass the “Comments to the Author” section, enter your conflict of interest statement in the “Confidential to Editor” section, and submit your "Accept" recommendation.

Reviewer #2: All comments have been addressed

Reviewer #5: All comments have been addressed

**********

2. Is the manuscript technically sound, and do the data support the conclusions?

The manuscript must describe a technically sound piece of scientific research with data that supports the conclusions. Experiments must have been conducted rigorously, with appropriate controls, replication, and sample sizes. The conclusions must be drawn appropriately based on the data presented. 

Reviewer #2: Yes

Reviewer #5: Yes

**********

3. Has the statistical analysis been performed appropriately and rigorously? 

Reviewer #2: (No Response)

Reviewer #5: Yes

**********

4. Have the authors made all data underlying the findings in their manuscript fully available?

The PLOS Data policy requires authors to make all data underlying the findings described in their manuscript fully available without restriction, with rare exception (please refer to the Data Availability Statement in the manuscript PDF file). The data should be provided as part of the manuscript or its supporting information, or deposited to a public repository. For example, in addition to summary statistics, the data points behind means, medians and variance measures should be available. If there are restrictions on publicly sharing data—e.g. participant privacy or use of data from a third party—those must be specified.

Reviewer #2: (No Response)

Reviewer #5: Yes

**********

5. Is the manuscript presented in an intelligible fashion and written in standard English?

PLOS ONE does not copyedit accepted manuscripts, so the language in submitted articles must be clear, correct, and unambiguous. Any typographical or grammatical errors should be corrected at revision, so please note any specific errors here.

Reviewer #2: (No Response)

Reviewer #5: Yes

**********

6. Review Comments to the Author

Please use the space provided to explain your answers to the questions above. You may also include additional comments for the author, including concerns about dual publication, research ethics, or publication ethics. (Please upload your review as an attachment if it exceeds 20,000 characters)

Reviewer #2: (No Response)

Reviewer #5: (No Response)

**********

7. PLOS authors have the option to publish the peer review history of their article (what does this mean?). If published, this will include your full peer review and any attached files.

If you choose “no”, your identity will remain anonymous but your review may still be made public.

**Do you want your identity to be public for this peer review?** For information about this choice, including consent withdrawal, please see our Privacy Policy.

Reviewer #2: No

Reviewer #5: No

**********

# Editor report: Acceptance letter

**DOI:** 10.1371/journal.pone.0294671.r006

**Authors:** Sathishkumar Veerappampalayam Easwaramoorthy

14 Nov 2023

PONE-D-23-07943R2 

Evaluating the cost of simplicity in score building: An example from alcohol research 

Dear Dr. Baggio:

I'm pleased to inform you that your manuscript has been deemed suitable for publication in PLOS ONE. Congratulations! Your manuscript is now with our production department. 

If your institution or institutions have a press office, please let them know about your upcoming paper now to help maximize its impact. If they'll be preparing press materials, please inform our press team within the next 48 hours. Your manuscript will remain under strict press embargo until 2 pm Eastern Time on the date of publication. For more information please contact onepress@plos.org.

If we can help with anything else, please email us at customercare@plos.org.

Thank you for submitting your work to PLOS ONE and supporting open access. 

Kind regards, 

PLOS ONE Editorial Office Staff

on behalf of

Dr. Sathishkumar Veerappampalayam Easwaramoorthy 

Academic Editor

PLOS ONE
